# Long-Term Visual Memory and Its Role in Learning Suppression

**DOI:** 10.3389/fpsyg.2018.01896

**Published:** 2018-10-12

**Authors:** Gabriel N. Friedman, Lance Johnson, Ziv M. Williams

**Affiliations:** ^1^Department of Neurosurgery, Harvard Medical School, Massachusetts General Hospital, Boston, MA, United States; ^2^Department of Neurobiology, Harvard University, Cambridge, MA, United States; ^3^Harvard-MIT Health Sciences and Technology, Boston, MA, United States; ^4^Program in Neuroscience, Harvard Medical School, Harvard University, Boston, MA, United States

**Keywords:** long-term memory, visual memory, learning suppression, visual search, memory consolidation

## Abstract

Long-term memory is a core aspect of human learning that permits a wide range of skills and behaviors often important for survival. While this core ability has been broadly observed for procedural and declarative memory, whether similar mechanisms subserve basic sensory or perceptual processes remains unclear. Here, we use a visual learning paradigm to show that training humans to search for common visual features in the environment leads to a persistent improvement in performance over consecutive days but, surprisingly, suppresses the subsequent ability to learn similar visual features. This suppression is reversed if the memory is prevented from consolidating, while still permitting the ability to learn multiple visual features simultaneously. These findings reveal a memory mechanism that may enable salient sensory patterns to persist in memory over prolonged durations, but which also functions to prevent false-positive detection by proactively suppressing new learning.

## Introduction

Learning is an essential aspect of human behavior (Squire, 2004). At its core, learning allows us to improve our performance in activities, such as playing the piano, over time but whether similar mechanisms of memory underlie other basic sensory processes remain unclear. It is well known that memory can take either implicit forms, which underlie procedural skills such as riding a bicycle, or explicit forms, which underlie the remembrance of factual content, such as being able to recall the year of one’s wedding anniversary (Schacter et al., 1993; Rugg et al., 1998). Visual search falls within the realm of implicit memory as it concerns a sensory process rather than a fact-based learning structure (Chun and Jiang, 1998). The extension of memory to the visual system is far less understood compared with other sensory or motor processes (Kristjánsson, 2000). Visual search is a task in which a participant must distinguish the identity and/or position of a “target” visual item from an array of “distractor” visual items (Sireteanu and Rettenbach, 1995). It is thought to be a primitive skill that allows us to rapidly identify visual features in our environment – from predators hiding in the bushes to a particular item of clothing (Yang and Zelinsky, 2009).

The process of visual search requires the coordination of multiple elements and processes including oculomotor control, covert visual attention, temporal integration of visual information, and memory of scene configuration (Eckstein, 2011). The components of visual search that are expected to be affected by training include oculomotor fixations and contralateral delay activity, an electrophysiological characteristic of visual working memory (Peterson et al., 2001, 2007; Emrich et al., 2009). Attentional systems are also known to play an important role in influencing search patterns during visual search (Jiang, 2018), while statistical learning is thought to influence how attention is distributed during visual search experiments (Failing and Theeuwes, 2018; Ferrante et al., 2018).

With repeated practice, this ability to identify specific contextual features can also improve over the course of a single training session (Eckstein, 2011). For example, it has been shown that repeated visual search can lead to improvement in performance over seconds to minutes (Võ and Wolfe, 2012) and that this increase in performance can be influenced by the presence or absence distractor visual items (Schneider and Fisk, 1982; Boettcher et al., 2013). It has also been found that repeated search for similarly positioned items can persist after training (Geyer et al., 2013), although questions remain to what extent this effect is dependent on memory for where visual items are presented in space as opposed to the extent that it is a memory for the visual items themselves (Jiang and Wagner, 2004; Travis et al., 2013).

These observations demonstrate that repeated search for specific visual features can improve with training, and also raise the intriguing possibility that this improvement could be further subserved by a form of long-term memory. In particular, if visual search is indeed modulated by memory processes, then we should expect that repeated training could lead to long-term improvement over consecutive days or perhaps be susceptible to phenomena observed in other forms of long-term memory such as consolidation, interference, and selectivity (Brady et al., 2008; Cunningham et al., 2015).

## Materials and Methods

**FIGURE 1:**
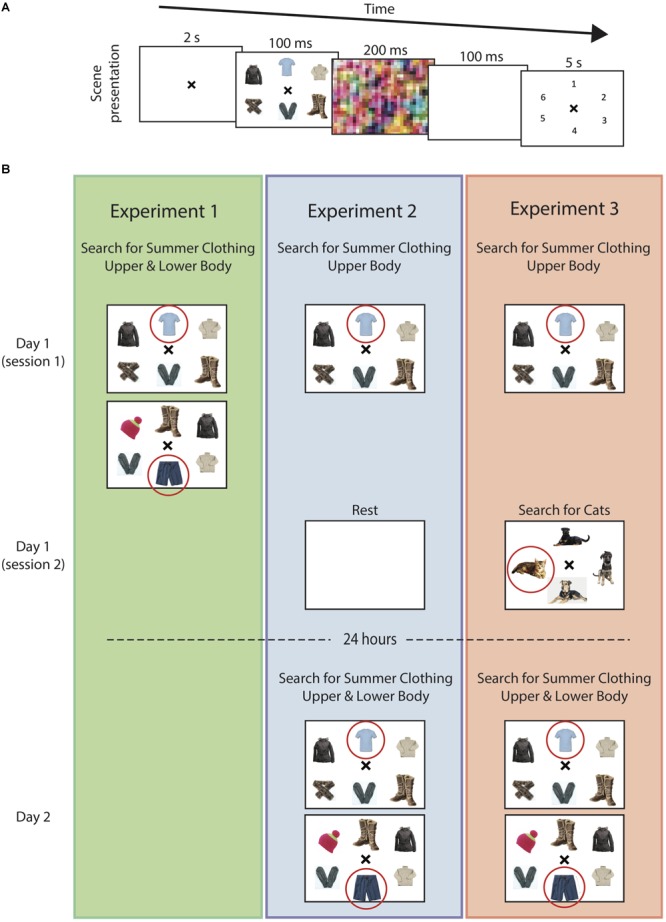
Visual search task. **(A)** In all experiments, subjects were shown an array of winter clothing items (distractors) with, in most cases, a single summer clothing item (target). Each array was followed by a visual screen. Subjects were then asked to select the location corresponding to the target search item. **(B)** In Experiment 1 (*left*), subjects searched for both upper-body and lower-body clothing items in randomly interspersed trials. In Experiment 2 (*center*), participants searched for upper-body clothing items on Day 1 and following 24 h, searched for upper-body and lower-body clothing items in randomly interspersed trials, similar to the paradigm used in Experiment 1. In Experiment 3 (*right*), participants searched for upper-body items on Day 1, similar to Experiment 2. Immediately following this experiment, subjects then performed an unrelated visual search task (cats vs. dogs). The following day, similar to Experiment 2, participants searched for both upper-body and lower-body clothing items in randomly interspersed trials. The circled item represents the target search summer clothing article (these were not shown during the task). Images were selected via with search engine provided at https://search.creativecommons.org.

Here, we devised a visual search paradigm that required participants to search for particular items from other similar items presented on a screen (representative arrays shown in **Figures 1A,B**). In order to distinguish between simple procedural improvements in response (due to task familiarity) and to evaluate the selectivity of visual long-term memory, two orthogonal categories of items were presented; (i) summer vs. winter clothing and (ii) upper vs. lower body clothing. Clothing items were chosen because of their validation in prior visual search experiments (Wolfe et al., 2000, 2007; Nako et al., 2014). The participants were always asked to identify the summer clothing item, as variations were made in whether summer/winter or upper/lower body clothing were presented and at what times over the course of the study they were given (Experiments 1–3).

Three main experiments were performed. For the first experiment, the participants were shown a slide with either a single upper-body or lower-body summer clothing item (e.g., short sleeve shirts, skirt, bathing suit, flip flop, etc.) that was positioned within an array of winter-clothing (e.g., sweaters, coats, scarves, gloves, etc.). For Experiment 1, the distractor items could come from either sub-category (i.e., a lower-body summer clothing item was surrounded by both upper- and lower-body winter clothing distractor items). The participants were only instructed to find the summer clothing item, without reference to whether the target item was an upper-body or lower-body clothing item. The upper- and lower-body summer clothing slides were randomly interspersed in order to assess baseline levels of task difficulty and learning patterns over the course of simultaneous training. The following two experiments were designed to assess for the presence of long-term visual memory and its selectivity. In Experiment 2, participants on the 1st day were asked to identify an upper-body summer-clothing item out of an array of winter clothing items. The next day, they had to identify either upper-body *or* lower-body summer clothing, again out of an array of winter clothing (i.e., the same as the first experiment but on Day 2). Experiment 3 was identical to Experiment 2 except that participants were given an interference task immediately following training in which they had to search for a new, unrelated set of items. For Experiments 2 and 3, distractor items on Day 1 were only upper-body clothing items, and upper- and lower-body items for Day 2 (consistent with the target items).

Experiment 1 was designed to evaluate baseline learning performances for the upper-body and lower-body search tasks. In contrast to the following two experiments, here, participants were asked to search for either upper- *or* lower-body summer clothing items on randomly interspersed trials throughout the experiment (**Figure 1B**). This allowed us to assess whether potential differences in learning over time could be due to a simple difference in baseline performance prior to learning or difficulty with learning two, within-category items, simultaneously. The participants performed a total of 88 trials over the course of this experiment.

Experiment 2 was designed to assess whether searching for items within a particular category can lead to sustained improvements in performance after 24 h, without intermediate training, and whether this improvement in performance was selective to the particular items being searched. As described above, the participants were first required to search for upper-body summer clothing from an array of winter clothing on Day 1 (**Figure 1**). To further examine the selectivity of training, on Day 1 the participants were given only an upper-body summer clothing item whereas on Day 2, after 24 h, they were given both upper- and lower-body summer clothing items (identical to Experiment 1). The participants performed a total of 88 such trials over the course of Day 1 and were then required to rest for 15 min after training without mental effort. After 24 h, they returned to complete the second search task.

Experiment 3 was used to examine whether the introduction of an interference task following training on Day 1 could influence the learning-suppression effect observed on Day 2 and further confirm that this effect is memory-selective. Here, the participants performed a similar set of trials as experiment 1, but now had to search for items within a different entry-level, rather than subordinate-level, category after training on Day 1 (**Figure 1**). The participants waited 15 min after finishing the initial visual memory task before starting the interference task.

The task was performed during mid-day so that it would have no interference with participants’ sleep-wake cycle, and participants were asked not to make adjustments to their normal daily routine. A total of 14 individuals participated in these experiments, with each participant only participating in one of the three experiments (ages 19–31, M:F ratio of 9:6). The experiments outlined in this study were found to meet the criteria for exemption by the Harvard Medical School Institutional Review Board. Therefore, an ethics approval was not required per our institution’s guidelines and United States federal regulations. Nevertheless, subjects provided their verbal informed consent prior to the initiation of their participation in this study.

For each experiment, the participants were presented with a screen containing the visual search task and were asked to identify a summer clothing item out of an array of otherwise winter clothing items. Each trial began with a blank screen and a central ‘x’ that provided a cue for the upcoming search task. Following cue presentation, the participants were presented with a random selection of items positioned in an equidistant circular configuration for 100 ms. On most trials, this arrangement constituted 1 article of summer clothing and 4–5 articles of winter clothing randomly positioned in circular configuration. These were then erased, and followed by the presentation of a visual mask. Finally, the participants were then given numbers at the same, previously presented locations, and were asked in which location, if any, they observed a piece of summer clothing (**Figure 1A**).

To limit guessing or a stereotypic spatial bias, 18% of trials contained no summer clothing and 31% of trials contained 4 rather than 5 upper-body winter clothing items. The items were randomly selected from a set of 19 target items (i.e., summer clothing) and 19 distractors (i.e., winter clothing), randomly positioned on the screen. If the participants did not see a piece of summer clothing, they were asked to select “none.” Finally, similar to prior reports (Potter et al., 2010), we measured performance in terms of accuracy in order to emphasize learning based on search rather than response reaction times which can be confounded by strategic spatial searching or variations in motoric performance between individuals (Cunningham and Wolfe, 2014).

### Statistical Analysis

The general approach taken in the data analysis was to assess differences in performance by using the paired *t*-test which allowed us to determine individual changes in performance, and by extension, memory, at the group level. Performance accuracy was quantified by tallying the participants responses with the correct target item location and dividing this number of correct responses by the total number of arrays visualized during the task. Experimental epochs were aggregated into time periods, the first half of an experiment and the second half, in order to assess changes in learning over the course of a single day, and to compare these changes on subsequent days. Data analysis was performed using MATLAB.

## Results

**FIGURE 2:**
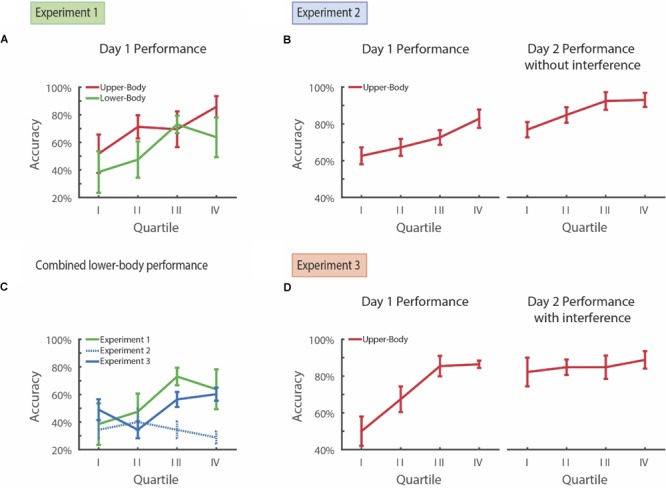
Long-term improvement in visual search after training and learning suppression.**(A)** Subjects performing simultaenous visual search for both upper- and lower-body in Experiment 1 demonstrate improvement in both overall and subcategory learning over the course of a single day. **(B)** Participants in Experiment 2 show learning over the course of Day 1 on upper-body visual search and this level of accuracy is maintained the following day for the upper-body visual search subcategory. **(C)** Subjects in Experiment 3, who performed the interference task on the prior day, demonstrated accuracy on the novel lower-body search task similar to subjects who participated in this task on Day 1 of Experiment 1. In contrast, subjects in Experiment 2, who did not perform the interference task on the prior day, showed significantly lower accuracy on the novel lower-body search task. **(D)** Subjects in Experiment 3 show improvement in upper-body learning during the course of Day 1 and, following an interference task on the 1st day, maintain this level of accuracy on the following day for the upper-body subcategory. Error bars represent ± 1 *SEM*.

In Experiment 1, participants demonstrated a significant improvement in performance over the course of training for both the lower-body (43% vs. 68%; paired *t*-test, *df* = 3, *d* = -1.60, *t*s = -3.20, *p* = 0.049) and upper-body visual search components (62% vs. 78%; paired *t*-test, *df* = 3, *d* = -1.91, *t*s = -3.82, *p* = 0.031; **Figure 2A**) when comparing the first- vs. second-half of the task. Taking these items together, the participants demonstrated significant learning over the course of training (52% vs. 75%; paired *t*-test, *df* = 3, *d* = -2.33, *t*s = -4.68, *p* = 0.019). There was no significant difference in performance for upper-body clothing and lower-body clothing on the second half of Day 1 (78% vs. 68%; paired *t*-test, *df* = 3, *d* = 0.30, *t*s = 0.77, *p* = 0.50).

Based on these data, we therefore conclude that (i) the participants were able to improve their performance over the course of training, (ii) they were able learn both the lower-body and upper-body items simultaneously, and (iii) there was relatively little difference in task difficulty between the lower- and upper-body items as evidenced by their final performances.

For Experiment 2, the participants demonstrated improvement in upper-body summer clothing search performance over the course of training on Day 1 (**Figure 2B**). Their performance increased from 65% in the first half of the training session to 78% on the second half of the training session (paired *t*-test, *df* = 4, *d* = -1.62, *t*s = -3.61, *p* = 0.023). While baseline performance varied somewhat between individuals, ranging from 50 to 75% within the first half of the session, all participants demonstrated an improvement in performance (**Supplementary Figure S1**).

Next, we tested whether improvement in performance persisted after 24 h without training. We first examined the Day 2 performance on the upper-body summer clothing search. Amongst the upper-body summer clothing presentations, the same participants from Day 1 had a similar performance on the first half of Day 2 as they had on the second half of Day 1 (81% vs. 78%; paired *t*-test, *df* = 4, *d* = -0.32, *t*s = -0.72, *p* = 0.51). Moreover, their performance on the first half of Day 2 was significantly higher than their performance on the first half of Day 1 (81% vs. 65%; paired *t*-test, *df* = 4, *d* = 1.62, *t*s = -4.12, *p* = 0.015). Their performance on Day 2 continued to slightly improve over the course of the session (93% vs. 81%; paired *t*-test, *df* = 4, *d* = 1.63, *t*s = -3.64, *p* = 0.022; **Figure 2B**). Therefore, training on Day 1 led to a sustained improvement in performance the following day.

Improvement in performance on Day 2 was *selective*, and did not simply result in an enhanced familiarity with the task or a generalized improvement in visual search. When we examined performance on the lower-body clothing search, which was new to these participants, their performance was 34% on the first half of Day 2. More notably, performance for lower-body clothing on the first half of Day 2 was both lower than first-half performance for upper body clothing on Day 2, at 81% (paired *t*-test, *df* = 4, *d* = -2.94, *t*s = -6.58, *p* = 0.0028), as well as lower than the 65% first-half accuracy of upper-body clothing search on Day 1 of Experiment 1 (paired *t*-test, *df* = 4, *d* = 1.6812, *t*s = -3.7592, *p* = 0.0198).

Improvement in performance on Day 2 appeared to *suppress* the ability to learn new lower-body items. Unlike the observed improvement in performance on Day 1 for the upper-body clothing, the participants were unable to improve their performance for the lower-body clothing over the course of Day 2. Specifically, they demonstrated no difference in performance between the first and second halves of Day 2 (37% vs. 32%; paired *t*-test, *df* = 4, *d* = 0.3987, *t*s = 0.89, *p* = 0.42). This difference was especially notable when comparing performances from experiment 1 and 2 (**Figure 2C**). Lower-body performance for the second half of Day 2 of Experiment 2 was also significantly lower than lower-body performance for the second half of Day 1 of Experiment 1 (32% vs. 68%; two-sample *t*-test, *df* = 4, *d* = -4.52, *t*s = 3.59, *p* = 0.0088). Moreover, as detailed above in Experiment 1, this lower performance for upper-body clothing was not simply due to a potential difficulty difference in searching for both upper- and lower-body clothing items simultaneously.

Taken together, these observations suggest that (i) training on the upper-body summer clothing on Day 1 limited the participant’s ability to improve their performance on lower-body summer clothing on Day 2, (ii) this lack of improvement was not due to difficulty in searching for upper- and lower-body items simultaneously, and (iii) prior learning of upper-body clothing did not generalize to improved performance on lower-body clothing as would be expected from task familiarity or simple procedural improvement.

For Experiment 3, similar to before, we find that these participants improved their performance for upper-body clothing items over the course of training on Day 1 (59 to 88%; paired *t*-test, *df* = 4, *d* = -2.96, *t*s = -6.63, *p* = 0.0027; **Figure 2D**). Here, however, immediately following training (i.e., after trial 88), they were then asked to search for pictures of cats (e.g., Tobe cat, Siamese cat, etc.) out of an array of dogs (as with the main task, some of trials contained no cats and about a third contained 4 rather than 5 dogs). Introduction of the interference task after searching for upper-body clothing on Day 1 *prevented* suppression of lower-body learning on Day 2. As in Experiment 1, overall upper-body search performance on Day 2 was significantly higher than lower-body search accuracy (84% vs. 50%; paired *t*-test, *df* = 4, *d* = 4.89, *t*s = -13.08, *p* = 0.00020). However, unlike Experiment 1, the participants in Experiment 3 demonstrated a significant improvement in performance on lower-body search over the course of the Day 2. Overall, their accuracy increased from 42 to 58% from the first half to the second half (paired *t*-test, *df* = 4, *d* = -1.51, *t*s = -3.38, *p* = 0.028). Lower-body performance for the second half of Day 2 of Experiment 3 was also significantly higher than that of lower-body performance for Experiment 2 (58% vs. 32%; two sample *t*-test, *df* = 4, *d* = 2.87, *t*s = 4.55, *p* = 0.0019). Therefore, interference on Day 1 following upper-body clothing training appeared to limit the suppressive effect of prior training on learning.

Improvement in performance for lower-body clothing in Experiment 3 was not due to a concurrent suppression of performance for upper-body clothing. Specifically, upper-body performance on the first half of Day 2 was 84% and was significantly higher than the 59% performance on the first half of Day 1 prior to interference (paired *t*-test, *df* = 4, *d* = 1.37, *t*s = -3.06, *p* = 0.038). Therefore, introduction of an interference task following training (i) did not lead to reduced performance for upper-body clothing the following day, but (ii) prevented the suppression of performance improvement for lower-body clothing.

**FIGURE 3:**
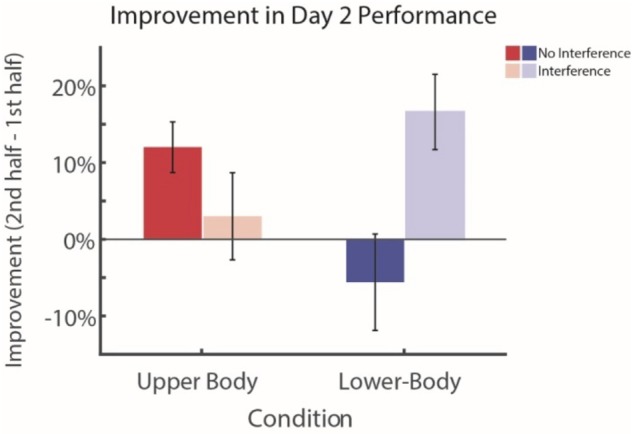
Effects of interference in long-term visual search performance and learning. Subjects who perform the interference task (Experiment 3) demonstrate significant improvement during the course of Day 2 for the novel lower-body visual search task. In contrast, subjects who do not perform the interference task (Experiment 2) demonstrate a significant decrease in learning on the novel lower-body search task, both when compared to their own improvement on the familiar upper-body search as well as in comparison to the learning of the interference group during lower-body visual search. Error bars represent ± 1 *SEM*.

Finally, we directly compared experiments 2 and 3 to evaluate the selectivity of interference on learning. We find that, when comparing the learning rates for Day 2 (difference between first and second half performance) of Experiment 2 to Experiment 3, participants who were given the interference task had a higher learning rate for the unfamiliar lower-body search task, compared to those who did not receive the interference task (two sample *t*-test, *df* = 4, *d* = -1.77, *t*s = -2.78, *p* = 0.024; see **Figure 3**). This suggested that both learning rate as well as *absolute* performance for those who received interference was higher for the novel items. By contrast, we did not observe this improvement for the group that did not receive interference, suggesting that the suppression from the previous task prevented learning from taking place. When comparing the performance solely of participants who did *not* receive interference (Experiment 2), they demonstrated significantly higher learning on the familiar upper-body task on day 2 compared with the unfamiliar lower-body task (two sample *t*-test, *df* = 4, *d* = 1.57, *t*s = 2.48, *p* = 0.038). Furthermore, these differences do not appear to be a result of differences in overall learning rates between the two groups, but rather specific to the individual upper- and lower-body clothing conditions (**Supplementary Figure S2**). We therefore conclude that the interference task prevented performance on the familiar items from suppressing improvement in performance for the novel items (see further below).

## Discussion

Humans and many animals have the ability to search for and rapidly identify complex sensory features in our environment such as the presence of predators or food items (Alexander and Zelinsky, 2011; Cunningham and Wolfe, 2014). This skill requires a rapid interplay between perceptual, attentional, executive function, and oculomotor systems (Eckstein, 2011), but there is mixed evidence regarding the extent to which memory processes subserve our visual search capabilities (Brady et al., 2011).

One model proposes that memory is not an integral element of visual search, either within a single trial (Horowitz and Wolfe, 1998), where performance benefits if non-target distractors can be ignored, or across trials, where implicit learning patterns could theoretically produce more efficient search (Wolfe et al., 2000). Moreover, there is some evidence that even in instances where short-term memory does develop during visual search, it does not provide an advantage over search that is based on simple visual feature analysis (Oliva et al., 2004). This line of research proposes an “amnesic” visual system that is “eternally present,” allowing individuals to purely focus their attention on specific visual features rather than relying on past memories to promote search ability (Horowitz and Wolfe, 1998).

Other models, on the other hand, have suggested a putative role for memory in the development of visual search. For example, some studies have shown that short-term memory can guide intra-trial search strategy (Gibson et al., 2000; Kristjánsson, 2000; Peterson et al., 2007; Höfler et al., 2015), and is affected by recent and delayed implicit priming effects (Kruijne and Meeter, 2016). Other studies have also shown that visual search produces short-term improvement in performance (Võ and Wolfe, 2012), and that visual search is susceptible to memory interference effects in the short-term (Schneider and Fisk, 1982; Boettcher et al., 2013) as well as associative links between both visually and semantically similar objects (Moores et al., 2003; Belke et al., 2008). Taken together, it seems likely that visual search is indeed influenced by short-term memory processes, but whether and by what processes such improvements may be represented over longer timescales has remained largely unknown.

Here, we find that searching for items of a particular category can improve performance in the long term. Specifically, we find that participants trained to search for a particular category of items can demonstrate a persistent improvement in performance without intermediate training over consecutive days. From that perspective, visual search training appears to produce a similar effect to that observed in other memory phenomena, such as item recognition and associative memory (Poldrack et al., 2001; Fell and Axmacher, 2011), whereby repeated training can produce a long lasting change in performance over two or more consecutive days.

However, we also find here that prior learning led to a suppression of performance when searching for novel subordinate-level items within the same category as well as to an inability to improve performance over time. These effects were not due to increased difficult or distraction as there was no suppression of performance when the items were initially learned concurrently. Moreover, introduction of an interference task following training did not lead to reduced performance for the initially trained item but rather prevented the suppression of performance improvement for the new items (i.e., it prevented learning suppression). While it could be argued that our study merely demonstrates new vs. familiar learning as opposed to true visual categorical learning, given that the distractors were context specific, it is unlikely that participants were simply gaining the ability to recognize individual visual search items.

Our interpretation of these findings is that searching for particular visual features within the environment can lead to long-term retention of the same visual features, but also suppresses the ability to form additional similar memory traces. These findings share some parallel with models of pattern suppression that may be category-modulated and potentially reversible with non-target category interference (Wimber et al., 2009, 2015; Maxcey and Woodman, 2014). However, they also demonstrate that prior learning prevents additional learning of unfamiliar categories. These findings are in contrast to prior observations that learning of categorical features can sometimes lead to improved categorization accuracy (Scott et al., 2008). Lastly, to further support this distinction between short- and long-term memory components of visual memory, interference after training on the original visual feature did not suppress performance when searching for that same feature the following day but prevented the suppression of additional visual features. Taken together, these data suggest a neural mechanism that may allow attended visual patterns to persist in memory over prolonged durations but which suppresses the ability to learn similar visual patterns once consolidation occurs. The prospective benefit of such a system could be to enhance specificity to trained visual features within the environment by suppressing the ability to learn similar but distinct visual features and, therefore, the possibility of ‘false-positive’ detection. These observations provide an important addition to our understanding of long-term memory processes (Frankland and Bontempi, 2005; Nader and Hardt, 2009).

## Data Availability Statement

The raw data supporting the conclusions of this manuscript will be made available by the authors, without undue reservation, to any qualified researcher.

## Author Contributions

GF, LJ, and ZW were involved in the conception and design of the study and conducted data analysis and interpretation. LJ and GF performed data collection. GF and ZW were involved in drafting the manuscript.

## Conflict of Interest Statement

The authors declare that the research was conducted in the absence of any commercial or financial relationships that could be construed as a potential conflict of interest.
