# Supplementary material for: Long-Term Visual Memory and Its Role in Learning Suppression
Source: Front Psychol. 2018 Oct 12;9:1896. doi: 10.3389/fpsyg.2018.01896 (PMC6194155; doi:10.3389/fpsyg.2018.01896)
Supplement: Supplementary file 1 [file Data_Sheet_1.docx]

Supplementary Material

**Long-term visual memory and its role in learning suppression**

Gabriel N. Friedman, Lance Johnson, Ziv M Williams

*** Correspondence:** Gabriel Friedman: gfriedman1@mgh.harvard.edu

Supplementary Figures

**
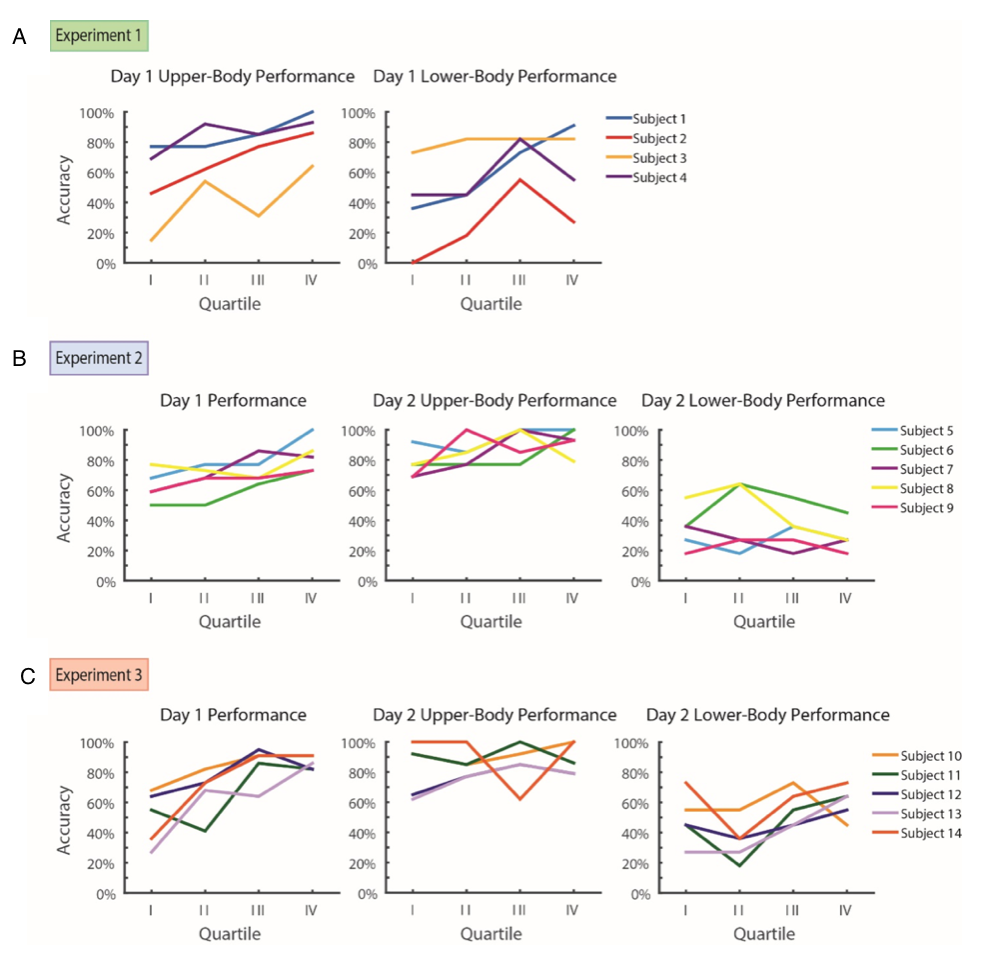
**

**Fig. S1**. **Individual performances across Experiments 1-3. A.** Individual subject data from Experiment 1 demonstrates improvements in performance across both upper-body and lower-body conditions. **B.** On Day 1 of Experiment 2, individual subjects improve on visual search performance and maintain this high level of accuracy on Day 2 on the previously trained upper-body visual search. However, lower-body search performance is notably less accurate. **C.** Similar to Experiment 2, subjects on Days 1 and 2 of Experiment 3 show improvement during the course of initial upper-body learning that is maintained the following day. However, in contrast to Experiment 2, individual subjects show improvement over the course of Day 2 lower-body visual search and have a higher accuracy for this portion compared with subjects from Experiment 2.


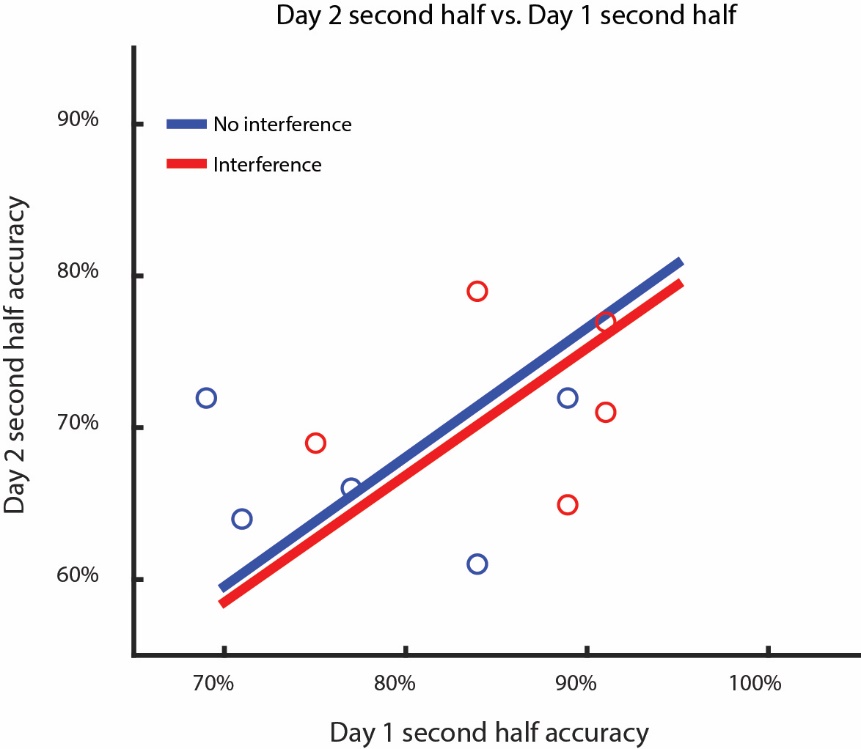


**Fig. S2. Correlation between visual search performances on Days 1 and 2 for individual subjects.** Subjects in both Experiment 2 and Experiment 3 showed similar levels of correlation between accuracy in their second half performance on Day 1 and Day 2, regardless of interference, suggesting that differences in pattern suppression are not due to differences in overall learning rates between subjects, but rather due to specific changes during the respective upper-body and lower-body learning conditions. Performance here is calculated for overall second half performance for both upper- and lower-body clothing.

**­­­­**
